# p16^INK4A^-deficiency predicts response to combined HER2 and CDK4/6 inhibition in HER2+ breast cancer brain metastases

**DOI:** 10.1038/s41467-022-29081-2

**Published:** 2022-03-18

**Authors:** Jing Ni, Sheheryar Kabraji, Shaozhen Xie, Yanzhi Wang, Peichen Pan, Xiaofang He, Zongming Liu, Jose Palbo Leone, Henry W. Long, Myles A. Brown, Eric P. Winer, Deborah A. R. Dillon, Nancy U. Lin, Jean J. Zhao

**Affiliations:** 1 Department of Cancer Biology, Dana-Farber Cancer Institute, Boston, MA 02215 USA; 2 Department of Biological Chemistry and Molecular Pharmacology, Harvard Medical School, Boston, MA 02115 USA; 3 Department of Medical Oncology, Dana-Farber Cancer Institute, Boston, MA 02215 USA; 4 Laboratory of Systems Pharmacology, Harvard Medical School, Boston, MA 02115 USA; 5 Center for Functional Cancer Epigenetics, Dana-Farber Cancer Institute, Boston, MA 02215 USA; 6 Department of Pathology, Brigham and Women’s Hospital, Boston, MA 02115 USA; 7 Present Address: College of Pharmaceutical Sciences, Zhejiang University, Hangzhou, Zhejiang 310058 P. R. China; 8 Present Address: Department of Radiation Oncology, the first affiliated hospital of Sun Yat-Sen University, Guangzhou, 510080 P. R. China; 9 Present Address: Key Laboratory of Bionic Engineering (Ministry of Education), College of Biological and Agricultural Engineering, Jilin University, Changchun, Jilin 130022 P. R. China; 10 Present Address: Department of Anesthesiology, Jilin Cancer Hospital, Changchun, Jilin 130000 P. R. China

**Keywords:** Breast cancer, Metastasis, Targeted therapies

## Abstract

Approximately 50% of patients with metastatic HER2-positive (HER2+) breast cancer develop brain metastases (BCBMs). We report that the tumor suppressor p16^INK4A^ is deficient in the majority of HER2+ BCBMs. p16^INK4A^-deficiency as measured by protein immunohistochemistry predicted response to combined tucatinib and abemaciclib in orthotopic patient-derived xenografts (PDXs) of HER2 + BCBMs. Our findings establish the rationale for a biomarker-driven clinical trial of combined CDK4/6- and HER2-targeted agents for patients with HER2 + BCBM.

## Abstract (web summary)

HER2+ breast cancer often develop brain metastases (BCBMs) that are difficult to treat. Here, the authors show that p16^INK4A^ loss in BCBMs from HER2+ breast tumors results in resistance to the HER2 inhibitor Tucatinib, and that CDK4/6 inhibition can restore sensitivity to this drug.

## Introduction

Breast cancer brain metastases (BCBM) cause significant morbidity and mortality and remain an important clinical challenge in treating breast cancer patients. Up to 50% of patients with metastatic human epidermal growth factor receptor 2 amplified (HER2+) breast cancer develop brain metastases and have a median survival of 18–36 months, despite multimodal therapy including surgery, radiation, and HER2-directed therapies^1,2^. Therefore, it is an unmet medical need to develop novel systemic therapies that are effective against HER2 + BCBMs^3^.

We previously showed that in extracranial models of HER2 + breast cancer, resistance to HER2-targeted therapy in HER2 + breast cancer is mediated by upregulation of cyclin D1 and CDK4, thus, susceptible to CDK4/6 inhibition^4^. We hypothesized that a combination of brain penetrating HER2-targeted drug and CDK4/6 inhibitor might constitute an effective therapy for *CDKN2A*/p16^INK4A^-deficient HER2 + BCBMs.

The *CDKN2A* gene locus, located on chromosome 9p21.3, is also referred to as p16^INK4A^, Multiple Tumor Suppressor 1 (MTS1) and p14^ARF,^^5–7^. It differentially encodes RNA transcripts for proteins p16^INK4A^ and, via an alternate open reading frame, p14^ARF^ (p19 in mice). p16^INK4A^ and p14^ARF^ have distinct functions. p14^ARF^ binds to MDM2, sequestering it in the nucleolus preventing its degradation of p53^8,9^. p16^INK4A^ binds to the CDK4 and CDK6, preventing their binding to cyclin D1 and thus inhibiting G1/S cell cycle progression by preventing RB hyperphosphorylation and activation of E2F-target genes^10^. For clarity, further discussion of *CDKN2A* DNA alterations and RNA expression will be referred to using the gene name *CKDN2A*, while discussion of the *CDKN2A* protein product p16^INK4A^ will be referred to by protein name, p16^INK4A^. Several lines of evidence support the role of *CDKN2A*/p16^INK4A^ as a tumor suppressor^11^. *CDKN2A* is one of the most commonly altered tumor suppressor genes across metastatic solid tumor types, ranging from 8.6% in breast cancer up to >70% of mesothelioma^12^. Loss of *CDKN2A*/p16^INK4A^ expression is both an early event in the transition from premalignant to malignant tumors as well as a late event in the transition from localized to metastatic disease^11^. Several mechanisms of inhibiting *CDKN2A* expression have been identified in tumors, including homozygous deletions, copy number loss, insertions/deletions, missense mutations, and promoter methylation^12^.

## Results

### p16^INK4A^-deficiency in a majority of HER2 + BCBM PDXs

**Fig. 1:**
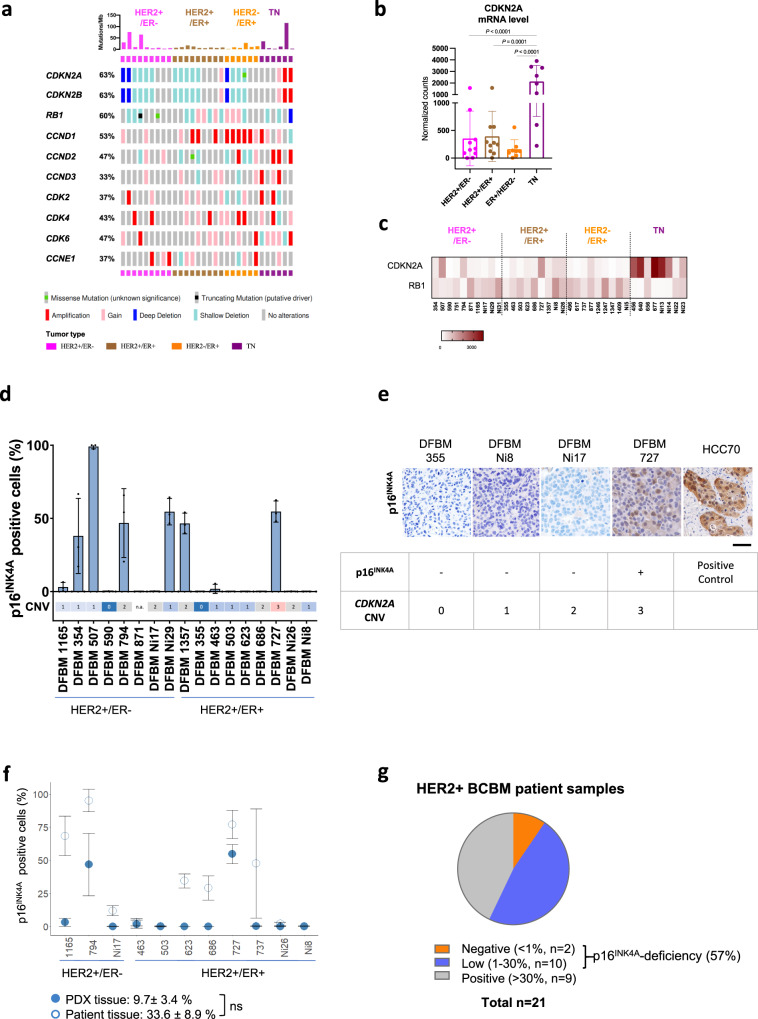
*CDKN2A* copy number variation and p16^INK4A^-deficiency are found in a majority of HER2 + BCBM PDXs. **a** Genomic analysis of genes associated with cell cycle pathway in BCBM PDXs. Major genetic alterations across 30 BCBM PDX samples. **b** *CDKN2A* mRNA expression levels across BCBM PDXs. Each dot represents one PDX model: HER2+/ER− (*n* = 10), HER2+/ER+ (*n* = 10), ER+/HER2− (*n* = 8), triple negative (TN) (*n* = 8), mean ± SD, one-way ANOVA followed by Tukey’s multiple comparisons test. **c** Heatmap of *CDKN2A* and *RB1* mRNA levels across BCBM PDXs. **d** Bar plot of p16^INK4A^ positive cells by immunohistochemistry (IHC) per HER2 + PDX model with *CDNK2A* copy number variation (CNV) shown below. p16^INK4A^ positive cells shown as mean ± SD of three images per section per sample. **e** p16^INK4A^ IHC of the indicated BCBM PDXs (Scale bar, 50 μm). HCC70 xenograft tumor sections were used as a positive control. **f** Comparison of p16^INK4A^ positive cells in HER2 + BCBM PDX and matched patient samples (*n* = 11, mean ± SD). Mean PDX p16^INK4A^ positive cells 9.7 ± 3.4%, mean patient p16^INK4A^ positive cells 33.6 ± 8.9%, *p* = 0.06. ns: not significant. **g** Proportion of patients in our BCBM cohort either negative or positive for p16^INK4A^ expression based on 30% cutoff. Source data are provided as a Source Data file.

To investigate BCBM-specific genetic alternations that mediate resistance to HER2-targeted inhibitors, we undertook genomic profiling of patient-derived xenografts of BCBMs, established as previously described^13^. Whole exome sequencing (WES) analysis of 30 PDXs (including 18 HER2+, 6 estrogen receptor-positive (ER+), and 6 triple-negative (TN) samples) revealed that genetic mutations and copy number variations found in major cancer genes are clustered in a number of main signaling pathways (Fig. 1a and Supplementary Fig. 1a). Notably, loss of *CDKN2A* and *CDKN2B* by copy number variation (CNV) is prevalent in HER2+ (11/18, independent of ER status) and ER+ (4/6), but not in TN BCBMs (Fig. 1a). RNA-seq analysis of these PDXs showed that *CDKN2A* mRNA expression was significantly lower in HER2+ or ER+ groups compared to that in TN groups (Fig. 1b). Moreover, the expression of *CDKN2A* mRNA appeared generally reciprocal with *RB1* mRNA, which has previously been observed in lung cancer cell lines and tissues (Fig. 1c)^14^. p16^INK4A^ and the retinoblastoma protein (RB) exist in a negative feedback loop such that in the absence of functional RB, p16^INK4A^ expression is increased^11,14^. This is a diagnostic feature of human papillomavirus (HPV) driven head and neck squamous cell carcinoma where expression of viral E7 protein results in RB degradation and reciprocal overexpression of p16^INK4A^ protein^15,16^.

Notably, while a majority of HER2 + BCBM PDXs have shallow (single copy) *CDKN2A* deletion (Fig. 1a), the expression levels of *CDKN2A* mRNA within the HER2 + group varied significantly (Fig. 1b, c). Thus, we developed a semi-automated quantitative immunohistochemistry (qIHC) pipeline to assess p16^INK4A^ protein expression in BCBM tissues from HER2 + PDXs and matched patient tissues (see Methods). We considered >30% p16^INK4A^ expression in cancer cells to be ‘positive’ for the purpose of this study. We found that p16^INK4A^ expression was low or undetectable in 11 out of 17 (64.7%) HER2 + BCBM PDX tissues (Fig. 1d, e). There is no significant difference in mean p16^INK4A^-positivity between PDX and matched patient tissue (mean PDX p16^INK4A^-positive cancer cells 9.7 ± 3.4%; mean patient p16^INK4A^-positive cancer cells 33.6 ± 8.9%, *p* = 0.06) (Fig. 1f). Numerical differences in mean p16^INK4A^ expression between matched patient and PDX tissues are likely due to intratumor heterogeneity and limitations of sampling when using single tissue sections for analysis. We confirmed that HER2 + BCBM PDX expression was well correlated with 11 matched patient tissues (*r* = 0.76, *p* = 0.003) (Supplementary Fig. 2a). Further analysis of our cohort of 21 HER2 + BCBM patient samples with p16^INK4A^ IHC revealed that 12/21 (57%) BCBMs were p16^INK4A^ deficient (<30% positive cells, Fig. 1g), consistent with our findings in the PDX models. Thus, these data suggest that a majority of HER2 + BCBMs have p16^INK4A^ deficiency. We extended our analysis of p16^INK4A^ expression to include HER2-negative subtypes and found that across breast cancer subtypes (ER+ HER2−, HER2+ and ER− HER2−), p16^INK4A^ expression is well correlated (*r* = 0.7, *p* = 1.6 × 10^−5^) between PDX and patient tissues (Supplementary Fig. 2b, c).

### Therapeutic response of p16^INK4^ negative HER2 + BCBM PDX to the combination of tucatinib and abemaciclib

Since *CDKN2A* CNV did not linearly predict for loss of p16^INK4A^ protein expression (Fig. 1d, e), we, therefore, used our panel of HER2 + PDXs BCBMs with varied CNV of *CDKN2A*, and p16^INK4A^ protein expression status (Fig. 1d, e), to test tucatinib, a brain penetrant HER2 inhibitor, and abemaciclib, a brain penetrant CDK4/6 inhibitor^17^, as monotherapy or combination.

**Fig. 2:**
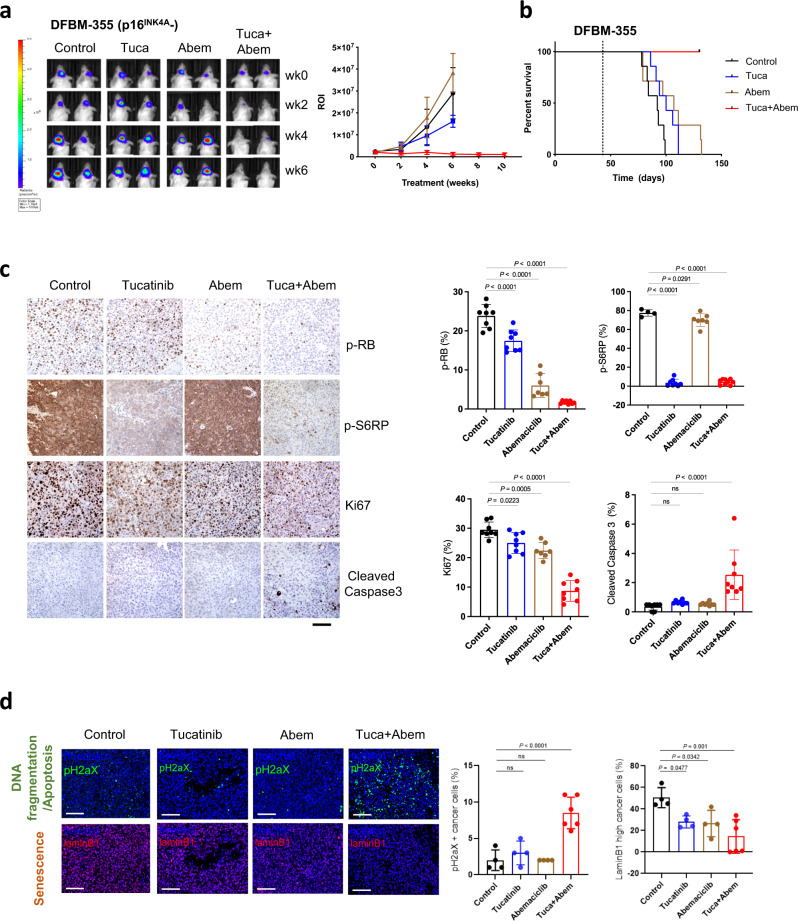
Therapeutic response of p16^INK4^ negative HER2 + BCBM PDX to the combination of tucatinib and abemaciclib. **a** Bioluminescence imaging analysis of mice bearing DFBM-355 tumors at indicated weeks after treatment with vehicle control, tucatinib (PO 75 mg/kg, BID), abemaciclib (Abem, PO 75 mg/kg, QD) or tucatinib and abemaciclib combination (Tuca + Abem). Representative bioluminescence images (left panel) and quantification (right panel) of the regions of interest (ROI) in each group of mice at indicated imaging time points. Mean ± SEM. Vehicle control (wk0,2,4, *n* = 7; wk6, *n* = 4), Tucatinib (wk0,2, n = 7; wk4, *n* = 6; wk6, *n* = 5), Abemaciclib (wk0,2, *n* = 7; wk4, *n* = 6; wk6, *n* = 3) or Tuca + Abem (wk0,2, *n* = 7; wk4, *n* = 6; wk6,8,10, *n* = 5). **b** Kaplan–Meier survival analysis of mice bearing DFBM-355 treated with vehicle control, tucatinib, abemaciclib, or Tuca + Abem as indicated. *n* = 7/group. The dotted line indicates the treatment starting time. **c** IHC analyses of p-RB (control, *n* = 8 fields; tucatinib, *n* = 8 fields; abemaciclib, *n* = 7 fields; Tuca + Abem, *n* = 8 fields), p-S6RP (control, *n* = 4 fields; tucatinib, *n* = 8 fields; abemaciclib, *n* = 7 fields; Tuca + Abem, *n* = 9 fields), Ki67 (control, *n* = 8 fields; tucatinib, *n* = 8 fields; abemaciclib, *n* = 7 fields; Tuca + Abem, *n* = 8 fields) and cleaved Caspase-3 (*n* = 8 fields/group) of DFBM-355 tumor samples harvested from tumor-bearing mice treated for 4 days with the same drugs at the same doses described in **a** (Scale bar, 100 μm). **d** Multiplex immunofluorescence assessment and quantification of change in apoptosis (as measured by pH2Ax) and senescence (as measured by Lamin B1) in DFBM-355. Representative 20x images are shown for each treatment condition. Scale bar, 100 μm. Four whole sections per condition were imaged except for Tuca + Abem where *n* = 6. For **c**, **d** mean ± SD. ns: not significant, One-way ANOVA followed by Dunnett’s multiple comparisons test. Source data are provided as a Source Data file.

We generated a cohort of mice bearing orthotopic tumors of DFBM-355, a HER2 + BCBM with *CDKN2A*-null and p16^INK4A^-deficiency (Fig. 1d, e), for the evaluation. Tucatinib or abemaciclib as monotherapy had little effect on the growth of orthotopic tumors and did not extend the survival of tumor-bearing mice (Fig. 2a, b). However, combined treatment with tucatinib and abemaciclib resulted in marked tumor regression examined by bioluminescence and significantly prolonged mice survival (Fig. 2a, b). To understand the mechanism underlying the robust effect of combining tucatinib with abemaciclib, we harvested tumors from tumor-bearing mice 4 days after treatment for pharmacodynamic (PD) assessment. As expected, abemaciclib markedly reduced the phosphorylation level of RB (p-RB), (which normally leads to inactivation of RB) indicating inhibition of cell cycle progression (Fig. 2c). Tucatinib treatment markedly decreased the phosphorylation level of S6RP (p-S6RP), a major signaling molecule downstream of the HER2/PI3K pathway (Fig. 2c)^13,18^. Consistent with the PD results, the combined treatment dramatically reduced cell proliferation (Ki67) and increased apoptosis (cleaved Caspase-3) (Fig. 2c). Using tissue-based highly multiplexed cyclic immunofluorescence (CyCIF), we further show that tucatinib plus abemaciclib had the strongest effect on reducing markers of cell cycle progression (Cyclin D1, phospho-RB, Geminin, phospho-Histone H3, Supplementary Fig. 3). We also assessed the expression of Lamin B1 and phospho-H2AX to assess whether cells were entering senescence or apoptosis^19,20^. We found the greatest reduction in Lamin B1 after treatment with tucatinb + abemaciclib (Fig. 2d)^21^. Notably, phospho-H2AX expression was significantly increased in tumors receiving tucatinib + abemaciclib compared to monotherapy (Fig. 2d). Because Lamin B1 expression can be lost by cells undergoing apoptosis as well as senescence, our analysis suggests that both mechanisms are likely responsible for efficacy of tucatinib + abemaciclib^22^. These results suggest that loss of *CDKN2A* and p16^INK4A^ renders HER2 + BCBM resistant to tucatinib, which can be overcome by addition of a CDK4/6 inhibitor.

**Fig. 3:**
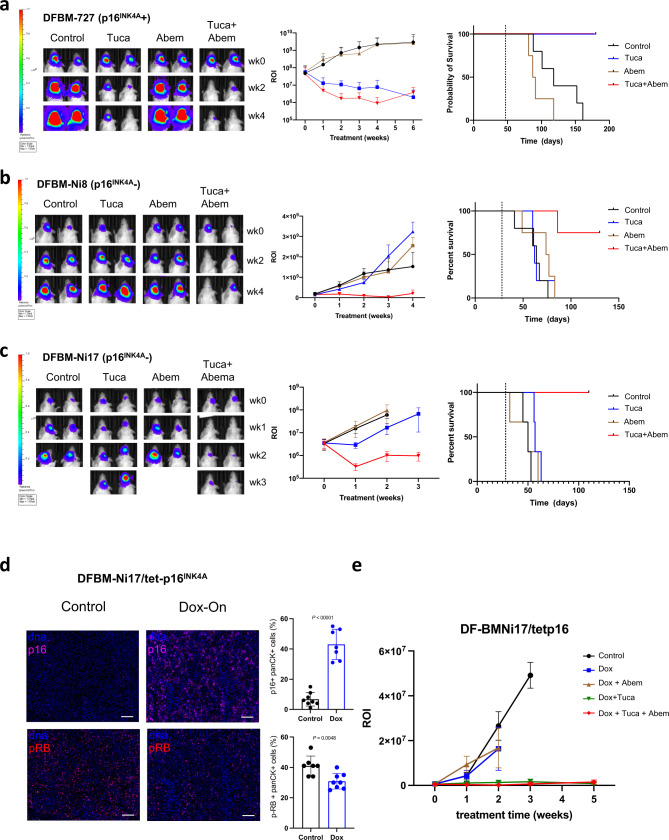
Loss of p16^INK4A^ is sufficient to predict response to tucatinib + abemaciclib in HER2 + BCBM PDX models. **a** Bioluminescence imaging (BLI) analysis with ROI of mice bearing DFBM-727 (p16^INK4A^ positive) tumors at indicated weeks after treatment with control, tucatinib (PO 75 mg/kg, BID), abemaciclib (PO 75 mg/kg, QD), or Tuca + Abem. Left panel: representative bioluminescence images. Middle panel: quantification of the regions of interest (ROI) in each group mice at indicated imaging time points, Vehicle control (*n* = 5), Tucatinib (*n* = 4), Abemaciclib (wk0-4, *n* = 4; wk6, *n* = 2) or Tuca + Abem (*n* = 5). Mean ± SD. Right panel: Kaplan–Meier survivals of mice bearing DFBM-727. Dotted lines indicate treatment starting time. **b** BLI and survival analysis for DFBM-Ni8 (p16^INK4A^ IHC negative, *CDKN2A* CNV = 1) treated as in **a**. BLI right panel: Vehicle control (wk0,1 *n* = 5; wk2,3, *n* = 4; wk4, *n* = 3), Tucatinib (*n* = 5), Abemaciclib (wk0-3, *n* = 4; wk4, *n* = 3) or Tuca + Abem (wk0-2, *n* = 5; wk3,4, *n* = 4). Mean ± SEM. **c** BLI and survival analysis for DFBM-Ni17 (p16^INK4A^ IHC negative, *CDKN2A* CNV = 2) treated as in **a**. BLI right panel: Vehicle control (*n* = 3), Tucatinib (*n* = 3), Abemaciclib (wk0, *n* = 3; wk1,2, *n* = 2), or Tuca + Abem (*n* = 3). Mean ± SEM. **d** Multiplex immunofluorescence shows increase in p16^INK4A^ (control, *n* = 8 sections; Dox, *n* = 7 sections) and reduction in p-RB (control, *n* = 7 sections; Dox, *n* = 8 sections) after 3 days of doxycycline administration to DFBM-Ni17/tetp16^INK4A^. Mean ± SD, Unpaired *t*-test. **e** BLI analysis with ROI of mice bearing DFBM-Ni17/tetp16^INK4A^ tumors at the indicated time with the treatment as indicated. Tucatinib (PO 75 mg/kg, BID), abemaciclib (PO 75 mg/kg, QD), doxycycline-diet (2500 ppm, ScottPharma). Control (*n* = 4), Dox (*n* = 4), Dox + Abem (*n* = 4), Dox + Tuc (wk0-3, *n* = 5; wk5, *n* = 4), Dox + Tuc + Abem (wk0-1, *n* = 5; wk2-5, *n* = 4). Mean ± SD. Source data are provided as a Source Data file.

We next evaluated DFBM-727, a HER2 + BCBM model with proficient *CDKN2A* CNV as well as p16^INK4A^ protein expression (Fig. 1f). Interestingly, DFBM-727 orthotopic tumors responded to tucatinib alone with prolonged survival of tumor-bearing mice (Fig. 3a). The addition of abemaciclib did not further enhance the effect of tucatinib on DFBM-727 in terms of tumor growth or mice survival (Fig. 3a). These data suggest that HER2 + BCBMs with proficient *CDKN2A* and p16^INK4A^ are sensitive to the brain penetrant HER2 inhibitor, tucatinib, consistent with its activity against HER2 + BCBM in the clinic^23^.

Given that DFBM-355 and DFBM-727 had responded differently based on their p16^INK4A^ protein expression status, we proceeded with testing two more p16^INK4A^-deficient HER2 + BCBM PDX models: DFBM-Ni8 (*CDKN2A* CNV 1) and DFBM-Ni17 (*CDKN2A* CNV 2) (Fig. 1f). Tucatinib or abemaciclib monotherapy had little effect on tumor growth and mice survival in either DFBM-Ni8 or DFBM-Ni17(Fig. 3b, c). However, combined treatment induced the tumor regression and maintained the stable disease in both of the models (Fig. 3b, c). These results indicate that p16^INK4A^ protein deficiency as measured by IHC, but not loss of heterozygosity as measured by *CDKN2A* CNV, predicts for response of HER2 + BCBMs to the combination of tucatinib and abemaciclib.

### Loss of p16^INK4A^ is sufficient to predict response to tucatinib + abemaciclib in HER2 + BCBM PDX models

**Fig. 4:**
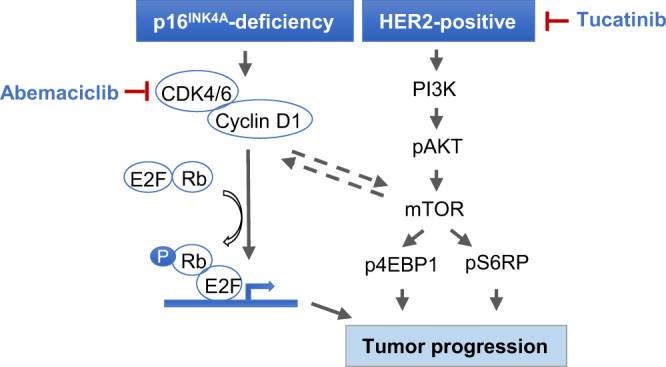
Schematic diagram of rational combination of tucatinib and abemaciclib for HER2+, p16^INK4A^-deficient BCBMs.

We next sought to further assess whether p16^INK4A^ status dictates the response of HER2 + BCBM to combined CDK4/6 inhibition with tucatinib. To establish a tumor model with restoring of p16^INK4A^ expression in an acute manner to avoid a potential complication of a long-term stable expression of p16^INK4A^-induced senescence, we restored p16^INK4A^ expression in DFBM-Ni17 tumor cells in an inducible fashion (referred as DFBM-Ni17/tetp16^INK4A^). We confirmed the expression of p16^INK4A^ in DFBM-Ni17/tetp16^INK4A^ tumors upon doxycycline administration to the tumor-bearing mice (Fig. 3d, Supplementary Fig. 4a). We also show that p16^INK4A^ expressing tumors had reduced levels of p-RB and proliferation without causing significantly increased senescence or apoptosis as compared to control tumors (Fig. 3d, Supplementary Fig. 4b–g). After 21 days post-intracranial transplantation of DFBM-Ni17/tetp16^INK4A^ BCBM tumor cells, these recipient mice were provided with doxycycline diet (dox) to induce p16^INK4A^ expression and started receiving tucatinib treatment. Restoring p16^INK4A^ expression in DFBM-Ni17 removes the need for CDK4/6 inhibitor to induce tumor response, so that tumors responded to tucatinib alone in the presence of p16^INK4A^ expression (Fig. 3e). These data suggest that loss of p16^INK4A^, the major endogenous CDK4/6 protein inhibitor, renders HER2 + BCBMs resistant to HER2-targeted therapy, which can be overcome by restoring p16^INK4A^ or a pharmacological small molecule inhibitor of CDK4/6 (Fig. 4).

## Discussion

Our data suggest that p16^INK4A^ protein deficiency as measured by quantitative IHC may be used as a biomarker to predict resistance to HER2-targeted monotherapy and response to the combination of tucatinib and abemaciclib in HER2 + BCBMs. We used a relatively conservative threshold of >30% p16^INK4A^ protein expression by IHC to p16 ^INK4A^ positive, recognizing that a higher threshold is used in other contexts^16^. p16^INK4A^ status may be relevant for predicting response to HER2 therapies in other solid tumors. In a recent study of patients with non-small cell lung cancer, 4/5 tumors with HER2-mutation were negative (expression score < 60) for p16^INK4A^ expression^24^. These data suggest that the combination of HER2 inhibition with CDK4/6 inhibition could be tested in patients with HER2 expressing tumors, stratified by loss of p16^INK4A^.

## Methods

### Patient-derived xenografts

Patient-derived xenografts were established by intracranial injection as described previously^13^. Briefly, informed consent was obtained from breast cancer patients and fresh brain metastases were then acquired from patients undergoing neurosurgery at the Brigham and Women’s Hospital, in accordance with Institutional Review Board (IRB) approved protocols (DFCI IRB 93-085 and 10–417). Patient tumor samples were dissociated with Collagenase/Hyaluronidase (Stemcell technologies) and Accutase (Sigma). Approximately 1e5 cells suspended in PBS (1–2 μl) was intracranially injected into 6–10 weeks old ICR-SCID female mice (Taconic, IcrTac:ICR-Prkdcscid) at the right striatum (2 mm to the right and 2.5 mm to the depth of the bregma). Mice were maintained on a 12-h dark/light cycle at ambient temperature (72 ± 2 F) with controlled humidity (~45%). All the animal experiments were performed according to protocols approved by the Dana-Farber Cancer Institute Animal Care and Use Committee in compliance with NIH animal guidelines.

### Whole exome sequencing

Whole exome sequencing was performed on Ion Proton platform (Thermo Fisher) and Ion S5 as described previously^13^. Key genes were selected for the signaling pathway using prior biological knowledge. For visualization of variants of these key genes, oncoprints were produced using an online tool OncoPrinter on http://cbioportal.org/oncoprinter^25,26^.

### RNA-seq

Total RNA was extracted by Trizol (Invitrogen) and RNeasy Mini Kit (Qiagen) following manual instruction. RNA concentration, purity, and RNA integrity number (RIN) were assessed using a NanoDrop ND-1000 spectrophotometer (NanoDrop, Wilmington, DE) and an Agilent 2100 Bioanalyzer (Agilent, Palo Alto, CA). RNA-seq sequencing was performed by Novogene on an Illumina Genome Analyzer (Illumina). Sequence data were processed through a bioinformatics pipeline for quality control and gene expression quantification (VIPER)^27^.

### Immunohistochemistry

Immunohistochemical (IHC) staining except p16^INK4A^ (see below) was performed as described previously^13,28^. Anti-Ki67 antibody (MIB-1, 1:200) was from DAKO. Anti-p-Rb (CST#8516, 1:400), anti-p-S6RP (CST#2211, 1:400), and anti-cleaved Caspase-3 (CST#9664, 1:400) antibodies were from Cell Signaling Technology. Data were collected with SPOT advanced version 4.6 or version 5.6 (SPOT imaging). Quantification of p-Rb, p-S6RP, Ki67, and cleaved Caspase-3 was conducted using the Image J software (version 1.50a).

p16^INK4A^ expression by chromogenic IHC in formalin-fixed paraffin-embedded (FFPE) PDX and patient tissues was determined using semi-quantitative image analysis. We stained for p16^INK4A^ by automated DAB(3,3′-Diaminobenzidine)-IHC with hematoxylin counterstain. Antibody against p16 ^INK4A^, from Ventana, catalog number 705–4793, clone E6H4 was run at 1:10 dilution using the Leica Biosystems Refine Detection Kit with EDTA antigen retrieval on the Leica Bond III automated staining platform. Three regions of interest (ROIs) per tumor section were manually selected for brightfield imaging at x40 to maximize tumor and exclude stroma where possible (Nikon). Standard exposure times were used for all images. One section was imaged per patient tissue or PDX model tissue. To determine percent positive cells, we first developed a CellProfiler (version 2.2)^29^ (www.cellprofiler.org) pipeline for automated detection of DAB-stained cells in a single section. A single version of this pipeline was used for all analysis and will be made available on cellprofiler.org/published-pipelines. A uniform threshold (0.3) was used across all samples to call hematoxylin positivity. In order to determine threshold for DAB positivity, eight test images from seven BCBM PDXs and one known p16^INK4A^ -positive cell line (HCC70), were scored for DAB-stained cells after p16^INK4A^ staining by three manual observers (SK, PP, DG). Each observer counted the number of DAB-stained cells and the number of hematoxylin-stained cells. A cell was called DAB positive if staining was above background in either nucleus or cytoplasm. Calling of DAB-stained cells was highly correlated between observers *r* > 0.75 (*p* < 0.03). We then tested the mean p16^INK4A^ positive cells called manually vs those called by our CellProfiler pipeline at three different DAB thresholds (0.15, 0.25, 0.3) using Bland Altman analysis. We chose a threshold of 0.3 because it represented the smallest bias compared to our manual observers (−13.19, (95% CI −357.6, 331.2)). Values shown are mean percent positive p16^INK4A^ cells ± SD per section, calculated from three ROIs. Percent positive cells = DAB positive cells/hematoxylin positive cells. p16^INK4A^ positivity of 30%, was the cutoff. Image raw data was analyzed using R software^30^ and figures were produced using the packages ggplot2 and ggpubr.

### Multiplex immunofluorescence microscopy

We used cyclic immunofluorescence to identify changes in cell cycle proteins and proliferation markers after treatment as previously described (www.cycif.org)^31,32^. Briefly, formalin-fixed paraffin-embedded BCBM PDX tissues were cut 5 um thick sections and underwent automated dewaxing, heated antigen retrieval using BOND autostainer (Leica). Tissues then underwent iterative staining, imaging, and bleaching with cell cycle antibodies (Supplementary Table 1). Imaging was performed on InCell Analyzer 6000 (GE) at 20x with uniform exposure for each channel. Images underwent stitching, registration, BaSIC correction using mcmicro (www.mcmicro.org)^33^. Quantification was performed in R and GraphPad Prism 9. Raw fluorescence intensity data were first normalized across all samples. Thresholds were determined by fitting a two-Gaussian mixture model to the normalized intensity data and the positive threshold was chosen to be peak of the positive distribution. Normalized values were then mapped back to raw values for calculation. Cell typing was performed according to the following rules. Tumor cells were identified by cytokeratin expression > 2.5. Tumor cells were then determined to be Lamin B1 positive if Lamin B1 > 3.3, cleaved Caspase-3 > 1.9. Tumor cells were determined to be pH2Ax positive if pH2Ax > 2.9. Thresholds for cell cycle markers were p16 > 2.8, p-RB > 2.7, PCNA > 3.3. Comparisons were made using *t*-test and the values shown are mean and standard deviation. Sample sizes are listed in the legend for each figure.

### In vivo drug treatment

For in vivo treatment, tucatinib (ONT380, Oncothyreon Inc) was dissolved in 5% NMP with 95% PEG300 and given oral twice daily at 75 mg/kg. Abemaciclib (Hoayuan ChemExpress Co. or TargetMol) was suspended in 5% NMP with 95% PEG300 and administered at 75 mg/kg daily by oral gavage. Mice were monitored daily.

### Bioluminescence imaging

Bioluminescence imaging was performed as previously described^34^. Briefly, mice bearing tumor with luciferase were injected IP with 80 mg/kg D-luciferin (Gold Biotechnology). After 10 min, bioluminescence signals were recorded with IVIS Lumina III Imaging System (Perkin Elmer) and analyzed with Living Image Software version 4.5 (Perkin Elmer).

### Western blot analysis

Samples were lysed and Western blotting was carried out as previously described^13,28^. Antibodies against p16^INK4A^ (Abcam ab108349, 1:1000) and α-tubulin (Signa #T9026, 1:5000) were applied. Data were recorded by Image Studio Lite version 5.2.5 (LI-COR).

### Overexpression of p16^INK4A^

DFBM-Ni17 cells were maintained in NeuroCult NS-A proliferation kit (Stemcell Technologies) supplemented with heparin (2 μg/mL), Epidermal Growth Factor (EGF, 20 ng/ml), and basic Fibroblast Growth Factor (bFGF, 10 ng/ml). Lentiviral production and transduction were performed as previously described^13,28^. Overexpression of p16^INK4A^ in cells were generated by infecting the cells with lentivirus encoding from pLX401-INK4A (Addgene #121919, doxycycline-inducible p16^INK4A^ expression). The cells were stably expressing inducible p16^INK4A^ by puromycin (1 μg/ml, EMD) selection. Doxycycline (1 μg/ml, PRI) or doxycycline diet (2500 ppm, ScottPharma Solutions) were applied to generate inducible p16^INK4A^ expression in vitro (cell culture) and in vivo (animal study).

### Statistical analysis

Statistical analyses were calculated using unpaired Student’s *t*-test or ANOVA by Prism 9 (GraphPad Software), excepted where otherwise stated. Pearson’s test of correlation was applied using R^30^. Data are considered significant when *P*-values are <0.05.

### Reporting summary

Further information on research design is available in the Nature Research Reporting Summary linked to this article.

## Supplementary information

Supplementary Information

Reporting Summary

## Data Availability

The Whole Exome Sequencing and RNA-seq data that support the findings of this study have been deposited in dbGAP with accession code # phs002482.v1.p1. All relevant data supporting the findings of this study are available in the manuscript and its supplementary information file and source data file. Source data are provided with this paper.

## Source data

Source data file
