# Supplementary material for: p16INK4A-deficiency predicts response to combined HER2 and CDK4/6 inhibition in HER2+ breast cancer brain metastases
Source: Nat Commun. 2022 Mar 18;13:1473. doi: 10.1038/s41467-022-29081-2 (PMC8933392; doi:10.1038/s41467-022-29081-2)
Supplement: Supplementary file 1 — Supplementary Information [file 41467_2022_29081_MOESM1_ESM.pdf]

Ni et al. Supplementary Information

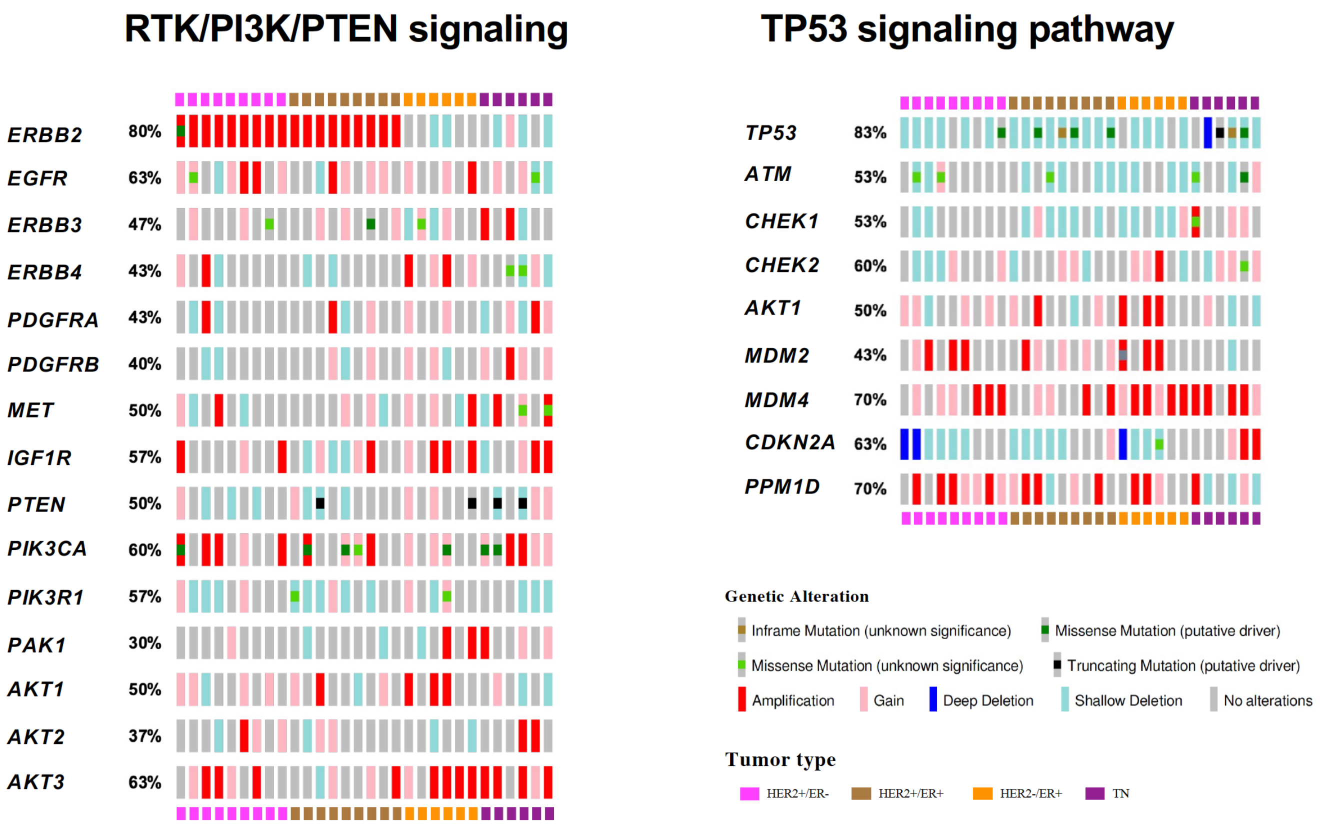

Supplementary Figure 1. Genomic analysis of RTK/PI3K/PTEN and TP53 signaling pathways in BCBM PDXs. Mutations and CNV status for each gene across 30 BCBM PDX samples are shown. Source data are provided as a Source Data file.

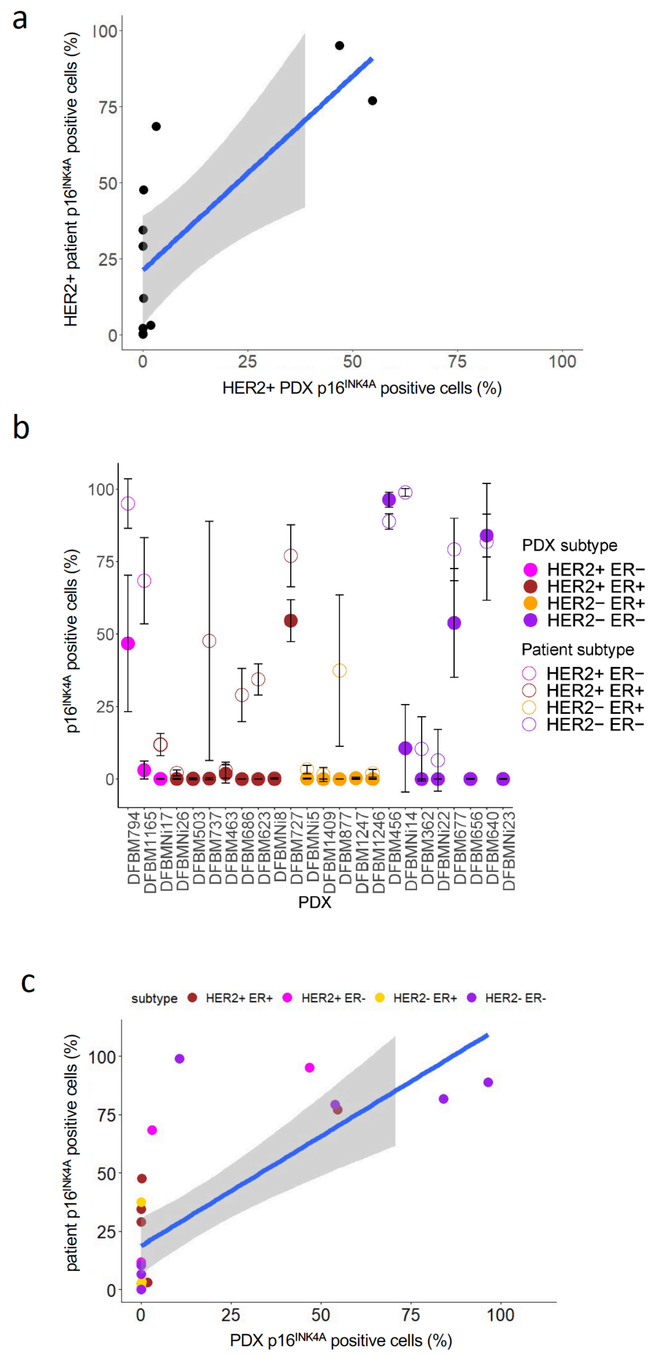

Supplementary Figure 2. (**a**) p16^{INK4A} positive cells in HER2+ BCBM PDX tissues are highly correlated with matched HER2+ BCBM patient tissues (n = 11, r = 0.76, p = 0.003, Pearson's test of correlation). (**b**) p16^{INK4A} expression by immunohistochemistry in BCBM PDX and matched patient tissues across all subtypes. Mean p16^{INK4A} positive cells in PDX tissues (solid circles) was not significantly different compared to matched patient tissues (empty circles). Data are presented as mean values ± SD. (14.7 ± 3.42% vs 32.5 ± 7.8 %, p = 0.07; n = 24). (**c**) Across all subtypes, p16^{INK4A} expression by immunohistochemistry in BCBM PDX and matched patient tissues is highly correlated (n = 24, r = 0.74, p = 1.6 x10^{-5}, Pearson's test of correlation). Source data are provided as a Source Data file.

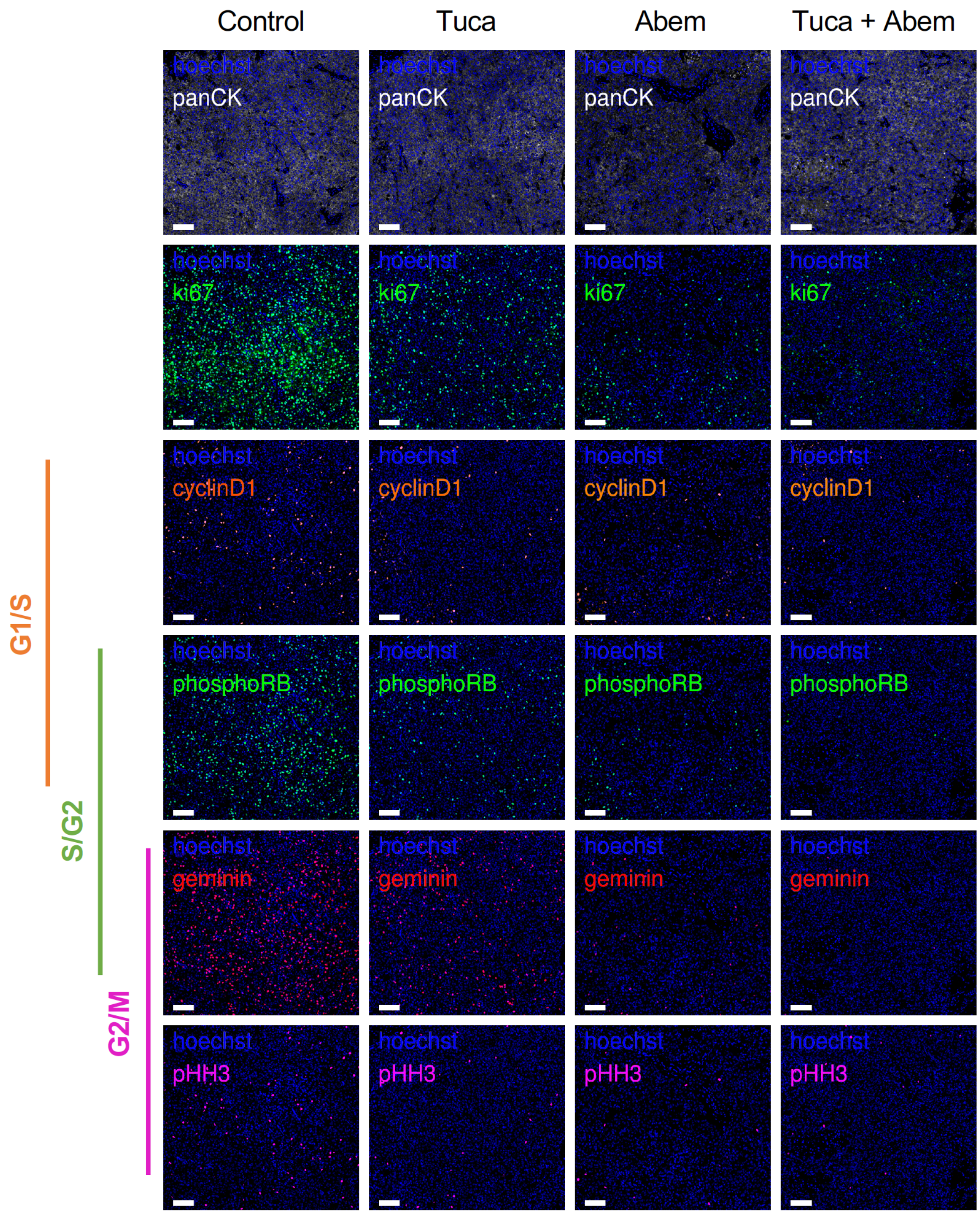

Supplementary Figure 3. Multiplexed tissue imaging of cell cycle and proliferation markers reveals that DFBM-355 treated with abemaciclib and tucatinib (as shown in **Figure 2a**) has reduced proliferation (as measured by Ki67) and loss of cell cycle marker expression across all phases (G1/S/G2) compared to monotherapy or control. Whole sections were imaged and representative fields shown. Scale bar = 100 μm.

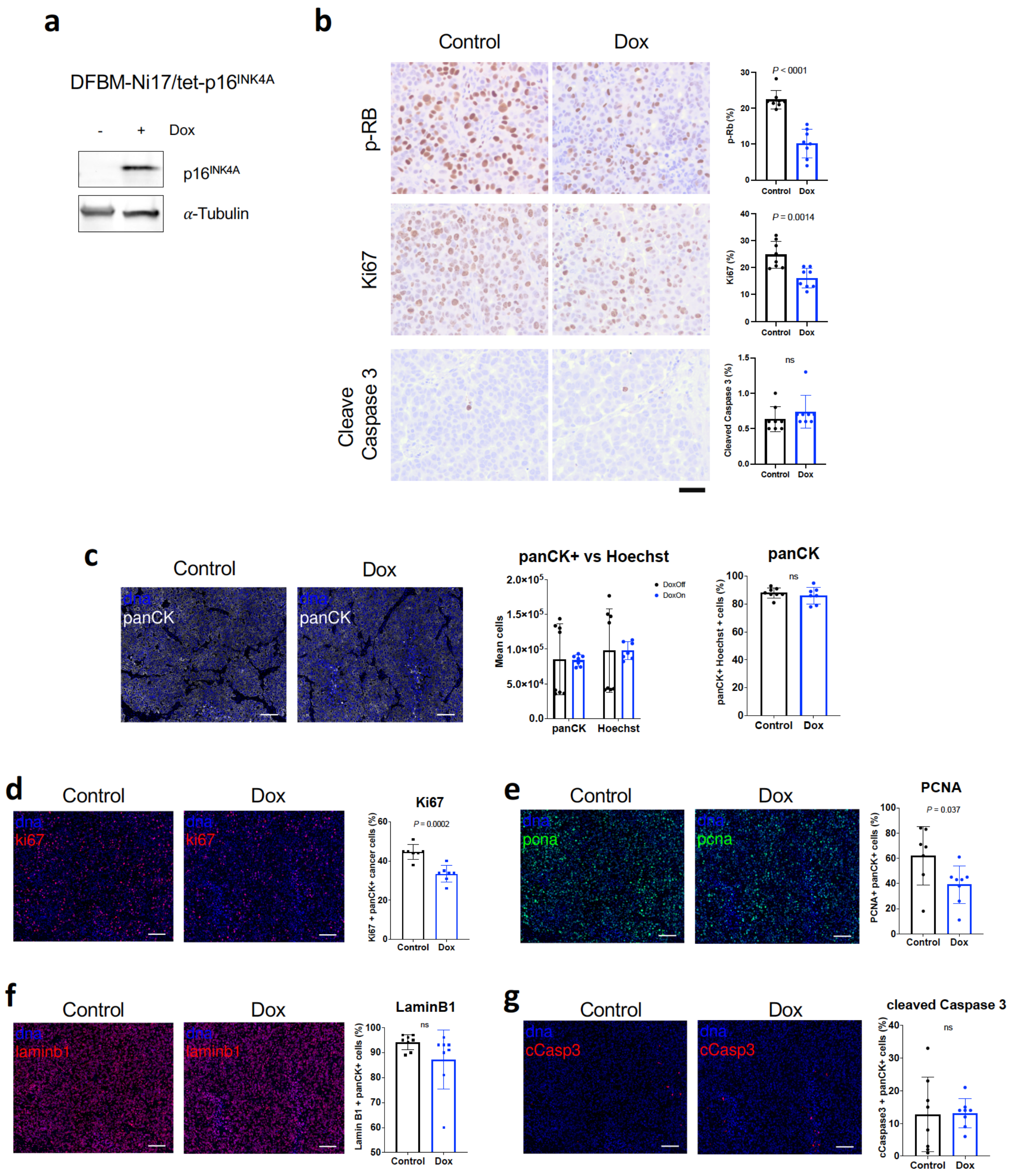

Supplementary Figure 4. (**a**) Western blot analysis of p16^{INK4A} expression in DFBM-Ni17/tetp16^{INK4A} cells. Cells were collected after 3 days of treatment with doxycycline (1 µg/ml) or vehicle control. Experiment was repeated twice, and representative data are shown. (**b**) Immunohistochemistry of p-RB, Ki67 and cleaved Caspase 3 of tumor tissues from DFBM-Ni17/tetp16^{INK4A} after 3 days of doxycycline administration. n = 8 /group. Scale bar, 50 µm. Multiplexed tissue imaging of (**c**) pan Cytokeratin (pan-CK, control, n = 8; Dox, n = 7), (**d,e**) proliferation (Ki67, n = 7/group; PCNA, control, n = 7; Dox, n = 8), (**f**) senescence (loss of Lamin B1, n

= 8/group) and (**g**) apoptosis (cleaved Caspase 3, n = 8/group) markers on DFBM-Ni17/tetp16^{INK4A} tumor tissues after 3 days of p16^{INK4A} induction. Scale bar 100 µm. For **b-g**, mean ± SD, Unpaired t-test. Source data are provided as a Source Data file.

Supplementary Table 1: List of antibodies used for multiplex immunofluorescence microscopy

Antibody Target	***Source***	***Catalog #***	***RRID***	***Standard Dilution***	***Clone Name***
Cyclin D1	Abcam	ab203448	AB_2890216	1:100	EPR2241
Geminin	Santa Cruz	sc-74456	AB_1124963	1:100	F7
Ki67	Cell Signaling Technology	11882S	AB_2687824	1:100	D3B5
Lamin B1	Abcam	ab194108	AB_2889226	1:100	EPR8985(B)
p16	Roche	705-4793	AB_2833232	1:10	E6H4
Pan-Cytokeratin	eBioscience	53-9003-82	AB_1834350	1:100	ae183
Phospho-Histone H2A.X	eBioscience	53-9865-82	AB_2574485	1:100	CR55T33
Phospho-Histone H3	Cell Signaling Technology	3475s	AB_10694639	1:100	D2C8
Phospho-RB	Cell Signaling Technology	4277S	AB_2797605	1:100	DB20B12
PCNA	Cell Signaling Technology	8580S	AB_11178664	1:100	PC10
