# Supplementary material for: p16INK4A-deficiency predicts response to combined HER2 and CDK4/6 inhibition in HER2+ breast cancer brain metastases
Source: Nat Commun. 2022 Mar 18;13:1473. doi: 10.1038/s41467-022-29081-2 (PMC8933392; doi:10.1038/s41467-022-29081-2)
Supplement: Supplementary file 2 — Reporting Summary [file 41467_2022_29081_MOESM2_ESM.pdf]

nature portfolio

Corresponding author(s): Jean Zhao

Last updated by author(s): Feb 14, 2022

Reporting Summary

Nature Portfolio wishes to improve the reproducibility of the work that we publish. This form provides structure for consistency and transparency in reporting. For further information on Nature Portfolio policies, see our Editorial Policies and the Editorial Policy Checklist.

Statistics

For all statistical analyses, confirm that the following items are present in the figure legend, table legend, main text, or Methods section.

n/a	Confirmed	
☐	☒	The exact sample size (*n*) for each experimental group/condition, given as a discrete number and unit of measurement
☐	☒	A statement on whether measurements were taken from distinct samples or whether the same sample was measured repeatedly
☐	☒	The statistical test(s) used AND whether they are one- or two-sided<br>*Only common tests should be described solely by name; describe more complex techniques in the Methods section.*
☒	☐	A description of all covariates tested
☐	☒	A description of any assumptions or corrections, such as tests of normality and adjustment for multiple comparisons
☐	☒	A full description of the statistical parameters including central tendency (e.g. means) or other basic estimates (e.g. regression coefficient) AND variation (e.g. standard deviation) or associated estimates of uncertainty (e.g. confidence intervals)
☐	☒	For null hypothesis testing, the test statistic (e.g. *F*, *t*, *r*) with confidence intervals, effect sizes, degrees of freedom and *P* value noted<br>*Give P values as exact values whenever suitable.*
☒	☐	For Bayesian analysis, information on the choice of priors and Markov chain Monte Carlo settings
☒	☐	For hierarchical and complex designs, identification of the appropriate level for tests and full reporting of outcomes
☐	☒	Estimates of effect sizes (e.g. Cohen's *d*, Pearson's *r*), indicating how they were calculated

Our web collection on statistics for biologists contains articles on many of the points above.

Software and code

Policy information about availability of computer code

Data collection	Ion Proton (Thermo Fisher), Prism version 9 (GraphPad), Living Image Software version 4.5 (PerkinElmer), SPOT advanced version 4.6 or version 5.6 (SPOT imaging), Image Studio Lite version 5.2.5 (LI-COR)
Data analysis	Ion Proton (Thermo Fisher), Prism version 9 (GraphPad), OncoPrinter (online tool, http://cbioportal.org/oncoprinter), Living Image Software version 4.5(PerkinElmer), Image J version 1.50a, CellProfiler version 2.2, R software with the packages ggplot2 and ggpubr

For manuscripts utilizing custom algorithms or software that are central to the research but not yet described in published literature, software must be made available to editors and reviewers. We strongly encourage code deposition in a community repository (e.g. GitHub). See the Nature Portfolio guidelines for submitting code & software for further information.

Data

Policy information about availability of data

All manuscripts must include a data availability statement. This statement should provide the following information, where applicable:

- Accession codes, unique identifiers, or web links for publicly available datasets
- A description of any restrictions on data availability
- For clinical datasets or third party data, please ensure that the statement adheres to our policy

The Whole Exome Sequencing and RNA-seq data that support the findings of this study have been deposited in dbGAP with accession code # phs002482.v1.p1. [https://www.ncbi.nlm.nih.gov/projects/gap/cgi-bin/study.cgi?study_id=phs002482.v1.p1]. All relevant data supporting the findings of this study are available in the manuscript and its supplementary information file and source data file.

Field-specific reporting

Please select the one below that is the best fit for your research. If you are not sure, read the appropriate sections before making your selection.

☒ Life sciences ☐ Behavioural & social sciences ☐ Ecological, evolutionary & environmental sciences

For a reference copy of the document with all sections, see nature.com/documents/nr-reporting-summary-flat.pdf

Life sciences study design

All studies must disclose on these points even when the disclosure is negative.

Sample size	For animal experiment, samples sizes were determined based on previous experience with the models utilized, including experience in variability of tumor growth (reference: Nat Med. 2016 Jul;22(7):723-6; Sci Rep. 2019 Jan 24; 9(1):622). Other sample sizes determined for experiments are included in the relevant figure legends.
Data exclusions	No data were excluded from analysis.
Replication	Experimental findings were reliably reproduced. We have included detailed numbers in the manuscript.
Randomization	Mice were randomized into groups of equal average tumor volume to the treatment groups.
Blinding	In vivo animal drug treatment, IHC experiment, and multiplex immunofluorescence microscopy with the outcome measurements and analysis were performed in a blinded fashion when possible. No other experiments required collection or quantification to be blinded.

Reporting for specific materials, systems and methods

We require information from authors about some types of materials, experimental systems and methods used in many studies. Here, indicate whether each material, system or method listed is relevant to your study. If you are not sure if a list item applies to your research, read the appropriate section before selecting a response.

Materials & experimental systems

n/a	Involved in the study	
☐	☒	Antibodies
☒	☐	Eukaryotic cell lines
☒	☐	Palaeontology and archaeology
☐	☒	Animals and other organisms
☐	☒	Human research participants
☒	☐	Clinical data
☒	☐	Dual use research of concern

Methods

n/a	Involved in the study	
☒	☐	ChIP-seq
☒	☐	Flow cytometry
☒	☐	MRI-based neuroimaging

Antibodies

Antibodies used

Antibodies for IHC:
Anti-Ki67 antibody (DAKO, MIB-1, 1:200)
Anti-p-Rb (Cell Signaling Technology, CST#8516, 1:400),
anti-p-S6RP (Cell Signaling Technology,CST#2211, 1:400)
anti-cleaved Caspase 3 (Cell Signaling Technology, CST#9664, 1:400)
Anti-p16 INK4A (Ventana, Cat#705-4793, clone E6H4, 1:10)

Antibodies for multiplex immunofluorescence microscopy
anti-Cyclin D1 (Abcam, ab203448, 1:100)
anti-Geminin (Santa Cruz, sc-74456, 1:100)
anti-Ki67 (Cell Signaling Technology, CST#11882S, 1:100)
anti-Lamin B1 (Abcam, ab194108, 1:100)
anti-p16 (Roche, #705-4793, 1:10)
anti-panCytokeratin (eBioscience, #53-9003-82, 1:100)
anti-p-Histone H2A.X (eBioscience, #53-9865-82, 1:100)
anti-Histone H3 (Cell Signaling Technology, CST#3475s, 1:100)
anti-p-RB (Cell Signaling Technology, CST#4277s, 1:100)
anti-cleaved Caspase 3 (Cell Signaling Technology, CST#9604s, 1:100)
anti-PCNA (Cell Signaling Technology, CST#8580s, 1:100)

Antibodies for Western blot:
anti-p16INK4A (Abcam, ab108349, 1:1000)

	anti-a-tubulin (Sigma, #T9026, 1:5000)
Validation	All the antibodies used in this study are commercially available and have been verified by the manufacturers according to the data on their websites.

Animals and other organisms

Policy information about studies involving animals; ARRIVE guidelines recommended for reporting animal research

Laboratory animals	ICR-SCID female mice (Taconic, IcrTac:ICR-Prkdcscid) were 6-10 weeks old at the beginning of the experiment. Mice were maintained on a 12-h dark/light cycle at ambient temperature (72 +/- 2F) with controlled humidity (~45%).
Wild animals	No wild animals were used in this study.
Field-collected samples	No field collections were used in this study.
Ethics oversight	All the animal experiments were performed according to protocols approved by the Dana-Farber Cancer Institute Animal Care and Use Committee in compliance with NIH animal guidelines.

Note that full information on the approval of the study protocol must also be provided in the manuscript.

Human research participants

Policy information about studies involving human research participants

Population characteristics	Patient-derived xenografts were derived from fresh breast cancer brain metastases (BCBM) acquired from patients undergoing neurosurgery at the Brigham and Women's Hospital<br>For IHC, human BCBM specimens were obtained from patients who underwent neurosurgery at the Brigham and Women's Hosptial
Recruitment	Informed consent was obtained from breast cancer patients and fresh brain metastases were then acquired from patients undergoing neurosurgery at the Brigham and Women's Hospital
Ethics oversight	Acquisition of human samples was approved by the Institutional Review Board (IRB) protocols (DFCI IRB 93-085, 10-417, 18-296)

Note that full information on the approval of the study protocol must also be provided in the manuscript.
